# Negative frequency dependent selection contributes to the maintenance of a global polymorphism in mitochondrial DNA

**DOI:** 10.1186/s12862-020-1581-2

**Published:** 2020-02-04

**Authors:** Zorana Kurbalija Novičić, Ahmed Sayadi, Mihailo Jelić, Göran Arnqvist

**Affiliations:** 1 Animal Ecology, Department of Ecology and Genetics, Evolutionary Biology Center, Uppsala University, Norbyvägen 18D, SE-752 36 Uppsala, Sweden; 2 Department of Neuroscience, Psychiatry, Uppsala University Hospital, Entrance 10, 751 85 Uppsala, Sweden; 3 Faculty of Biology, University of Belgrade, Studentski trg 16, Belgrade, 11000 Serbia

**Keywords:** Balancing selection, Mitochondria, mtDNA, Polymorphism, Negative frequency dependent selection

## Abstract

**Background:**

Understanding the forces that maintain diversity across a range of scales is at the very heart of biology. Frequency-dependent processes are generally recognized as the most central process for the maintenance of ecological diversity. The same is, however, not generally true for genetic diversity. Negative frequency dependent selection, where rare genotypes have an advantage, is often regarded as a relatively weak force in maintaining genetic variation in life history traits because recombination disassociates alleles across many genes. Yet, many regions of the genome show low rates of recombination and genetic variation in such regions (i.e., supergenes) may in theory be upheld by frequency dependent selection.

**Results:**

We studied what is essentially a ubiquitous life history supergene (i.e., mitochondrial DNA) in the fruit fly *Drosophila subobscura*, showing sympatric polymorphism with two main mtDNA genotypes co-occurring in populations world-wide. Using an experimental evolution approach involving manipulations of genotype starting frequencies, we show that negative frequency dependent selection indeed acts to maintain genetic variation in this region. Moreover, the strength of selection was affected by food resource conditions.

**Conclusions:**

Our work provides novel experimental support for the view that balancing selection through negative frequency dependency acts to maintain genetic variation in life history genes. We suggest that the emergence of negative frequency dependent selection on mtDNA is symptomatic of the fundamental link between ecological processes related to resource use and the maintenance of genetic variation.

## Background

Species diversity within ecological communities, which should erode over time as a result of stochastic events, competitive exclusion and unstable host-enemy dynamics, is to a large extent maintained by various mechanisms that generate negative frequency-dependent feed-back whereby rarer species are favored [1, 2]. Similarly, genetic polymorphism should be eroded by natural selection and genetic drift [3] and it has long been recognized that negative frequency-dependent selection (NFDS), where rare variants have a selective advantage over more common ones, is in theory the most powerful processes capable of maintaining genetic polymorphism within populations and species [4, 5]. Yet, while frequency-dependency has a central position in ecology, this is not true to the same extent in population genetics [2].

Lewontin [5] suggested that NFDS should quite generally act to maintain polymorphism whenever there is (i) temporal or spatial environmental variation, (ii) density-dependent resource competition and (iii) G × E interactions, conditions which should be the rule rather than the exception in natural populations [6]. In fact, theory suggests that frequency-dependent selection is an emergent and general property of density-dependency whenever competition is asymmetric [7, 8] such that, in essence, the most severe competitor for a given genotype is itself. Broadly defined, NFDS can result from inherent properties of specific genotypes or be an emergent result of density-dependent processes and environmental heterogeneity in space or time [6, 9]. However, because recombination disassociates combinations between alleles at multiple loci, such that a given individual may carry a mix of common and rare alleles across many genes, NFDS is often regarded as a weak force in maintaining polymorphism in major fitness components such as polygenic life history traits. Empirical efforts to understand NFDS have therefore been largely devoted to specific traits or genes with major effects [10], such as self-incompatibility in plants [11], immunity genes in vertebrates [12], rare male mating advantage [13], sexually dimorphic signaling [14, 15], mimicry [16] and anti-predatory coloration [17]. However, recent studies have shown that recombination rate varies markedly across the genome and linked sites sometimes tend to co-segregate as blocks, i.e. supergenes, for example as a result of chromosomal inversions [18]. Detecting NFDS is very challenging [10, 19] but several studies have implied a more general role for NFDS in cases where recombination is reduced, such as in bacteria [20] and in species where key phenotypes are affected by inversions [21–23]. The fact that supergenes can have major effects on variation in life history traits [21, 24, 25];) supports the possibility NFDS may contribute to the maintenance of polymorphism also in life history genes [4, 26, 27].

**Fig. 1:**
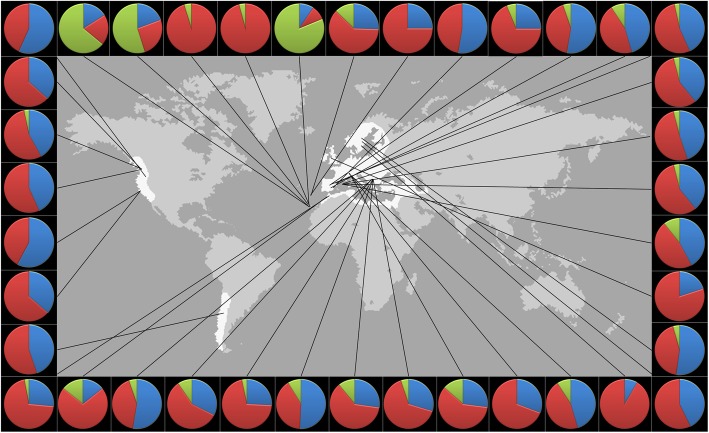
Mitochondrial DNA variation within natural populations of *D. subobscura* is heavily dominated by the two haplotype groups HI (blue) and HII (red) in all three disjunct parts of its global distribution (white). Apart from populations on the Canary Islands, where other haplotypes (green) are common, the mtDNA haplotype frequencies are similar across populations, with on average 37% of all individuals carrying HI and 58% HII haplotypes. This observation alone suggests that balancing selection is contributing to the maintenance of genetic variation in mtDNA. Several experimental studies have shown that flies carrying HI and HII differ in key life history traits, such as as metabolic rate, development time, adult longevity and desiccation resistance. Distribution map reproduced with permission from Rodríguez-Trelles et al. [42]

Here, we ask whether the classic, well characterized and striking pattern of polymorphism in mtDNA within natural populations of the fruit fly *Drosophila subobscura* (see Fig. 1) is the result of NFDS. Mitochondrial DNA (mtDNA) can be regarded as a life history supergene [24], which is maternally inherited, haploid and carries 13 protein coding genes which co-segregate with several sites that affect mitochondrial transcription and translation [28]. The mtDNA genes encode for parts of the ATP-producing OXPHOS pathway of the inner mitochondrial membrane and are as such key metabolic genes and important candidate genes for rate-dependent life history traits in eukaryotes [24, 29]. Segregating mitochondrial DNA haplotypes were long assumed to be functionally equivalent and thus neutral, but this view has changed markedly during the last decade (e.g., [24, 30, 31]). In *D. subobscura*, for example, a number of previous studies have shown that individuals carrying the two major mtDNA haplotypes HI and HII show differences in a range of key life history traits, such as metabolic rate [32], sex-specific juvenile survival [33], male fertility [34], development time [35], adult longevity [33, 35], desiccation resistance [35] and in estimates of fitness [35–37]. A few non-experimental studies of other insects [38–40], as well as an experimental study of non-sympatric haplotypes [41], have indicated that segregating mtDNA variation may be maintained by NFDS but experimental evidence for this is currently lacking.

We initiated a set of experimental evolution lines (*N* = 12), using a 2 × 2 crossed design, aimed at experimentally testing whether NFDS acts on sympatric mtDNA haplotypes of *D. subobscura* and, in addition, to assess whether resource conditions affect NFDS. Previous non-experimental work in this species has shown that mtDNA haplotype frequencies are polymorphic and stable over time in cage cultures, in a manner consistent with NFDS [38]. Our laboratory populations were (i) founded by contrasting starting frequencies of the two dominating mtDNA haplotype groups HI and HII (20/80% vs. 80/20%) (Fig. 1) and were (ii) allowed to evolve under competitive conditions in a more or less homogenous environment with regards to food resource quality. Flies used were from mitonuclear introgression lines constructed from a single natural population and all flies used share the same outbred nuclear genetic background, and were allowed to evolve in the laboratory for 10 non-overlapping generations. We sequenced and assembled the mitogenomes used (three HI and three HII; Additional file 1) and then employed PoolSeq to estimate genotype frequencies in samples of flies from all laboratory populations at generation 5 and 10. We make two explicit predictions. Most importantly, we predict that haplotypes should tend to increase in frequency when rare and decrease when common if NFDS contributes to the maintenance of polymorphism for the HI and HII haplotype groups. Moreover, if NFDS results from resource competition and G × E interactions [5], then the strength of NFDS should be affected by our food resource treatment.

## Results

**Fig. 2:**
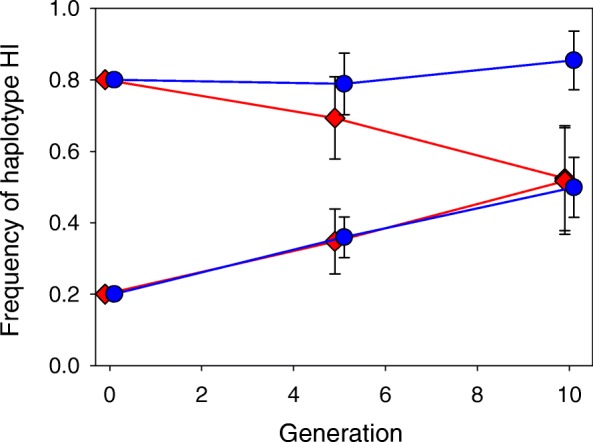
Replicated lines of *D. subobscura* were founded with flies carrying either mtDNA haplotype HI or HII in different proportions. This figure shows the mean frequency (±SE) of haplotype HI within lines during the course of experimental evolution. Haplotype I increased in frequency when rare and decreased in frequency when common, providing evidence for overall negative frequency dependent selection (red: homogenous resource environment; blue: heterogeneous resource environment)

**Fig. 3:**
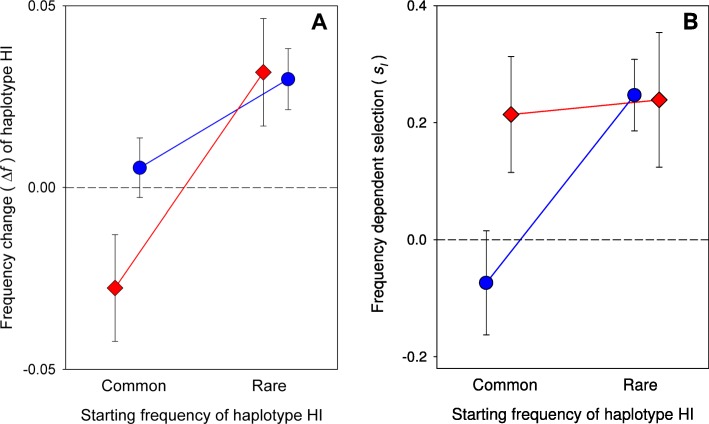
(**a**) Per generation changes in the frequency of HI during 10 generations of experimental evolution, when HI was started as being either the more common or the rarer haplotype. (**b**) The estimated strength of frequency dependent selection (*S*_*I*_) on mtDNA haplotypes over 10 generations, where positive values correspond to negative frequency dependency. Selection on mtDNA haplotypes was overall negatively frequency dependent, although those lines where HI was initially rare and that simultaneously experienced a more heterogeneous resource environment showed little evidence of selection. Given here are mean values (±SE) across lines (red: homogenous environment; blue: heterogeneous environment)

The key test for NFDS is one showing that the relative fitness of a genotype is inversely proportional to its frequency. As suggested by the general pattern of genotype frequency shifts during the experiment (Fig. 2), there was indeed an overall significant effect of starting frequency on mtDNA haplotype frequency changes (Table 1). The mean frequency of haplotype HI increased significantly in frequency when rare and decreased when common, and this was consistent over the course of our experiment (Fig. 3a). While the effects of starting frequency on frequency changes seemed weaker in the more heterogeneous resource environment, an overall effect of resource conditions on frequency change was not supported by these analyses (Table 1; non-significant SF × E interaction).

**Table 1:** Repeated measures ANOVAs of the effects of starting frequency (rare or common) and environmental conditions (homogenous or heterogeneous) on changes in the frequency of mtDNA haplotype HI and the strength of frequency dependent selection in the experimental evolution lines. Generation represents the within-line effect of generation 0–5 versus 5–10

	Frequency change of HI (Δ*f*)					Frequency dependent selection coefficient (*S*_*I*_)				
Between-line effects:	df	MS	*F*	*P*	*P*_perm_^a^	df	MS	*F*	*P*	*P*_perm_^a^
Starting frequency (SF)	**1**	**1.1E-02**	**8.091**	**0.022**	**0.021**	1	2.1E-02	0.048	0.832	0.849
Environment (E)	1	1.5E-03	1.123	0.320	0.336	1	4.5E-02	0.104	0.756	0.786
SF × E	1	1.8E-03	1.402	0.270	0.276	1	**2.3E+ 00**	**5.394**	**0.049**	**0.034**
Residual	8	1.3E-03				8	4.3E-01			
Within-line effects:										
Generation (G)	1	4.5E-06	0.001	0.972		1	2.1E-01	0.308	0.594	
G × SF	1	2.4E-06	0.001	0.979		1	4.0E-01	0.585	0.466	
G × E	1	1.4E-04	0.043	0.842		1	1.5E-01	0.214	0.656	
G × SF × E	1	4.9E-04	0.146	0.713		1	1.6E+ 00	2.348	0.164	
Residual	8	3.4E-03				8	6.8E-01			

^a^ Permutation tests of between-line effects

Both entries in bold face are significant at alpha = 0.05

Analyses of selection coefficients derived from our data provided further support for our main prediction of overall NFDS across our 12 laboratory populations, as the estimate of mean *S*_I_ was larger than zero (global *t*-test of H_0_: *S*_I_ = 0, *t*_11_ = 2.47, *P* = 0.031; bootstrap mean *S*_I_ = 0.16, 95% CI: 0.03–0.27). In contrast to the analyses of haplotype frequency changes, however, the analyses of selection coefficients also provided support for the effect of starting frequency on selection being contingent upon the environmental conditions (Table 1; significant SF × E interaction). The latter effect was largely due to an apparent absence of selection in one of our four treatment groups (Fig. 3b), where lines showed little change in mean haplotype frequency over time (Fig. 3a), rendering NFDS to be stronger overall in the homogenous compared to the heterogeneous resource environments. A closer inspection of the data supported this interpretation. Mean *S*_I_ was non-zero under homogenous food conditions when HI was started as both common (bootstrap mean *S*_I_ = 0.22, 95% CI: 0.09–0.34) and rare (bootstrap mean *S*_I_ = 0.24, 95% CI: 0.09–0.39). In contrast, mean *S*_I_ was non-zero under heterogeneous food conditions when HI was started as rare (bootstrap mean *S*_I_ = 0.25, 95% CI: 0.16–0.33) but not when started as common (bootstrap mean *S*_I_ = − 0.07, 95% CI: − 0.19 – 0.05).

Average population size during the experimental evolution was *N* = 773 (SE = 56) individuals. Population size increased markedly during the first five generations of our experiment and grew more rapidly to a larger size in populations experiencing the more heterogeneous environment (Additional file 1).

## Discussion

Our main results provide direct experimental support for the hypothesis that a classic and enigmatic genetic polymorphism (Fig. 1) in what is essentially a general life history supergene [24] is at least partly attributable to NFDS, as the relative fitness of haplotypes was inversely proportional to their starting frequency in our experimental evolution lines. While most classic studies of NFDS in other regions of the genome involve quite specific biological mechanisms [10], our findings are consistent with a more general role for NFDS. Life history strategies represent multidimensional phenotypes, characterized by differences in growth, reproduction and ageing, and it is likely that the relative fitness of a given life history strategy frequently and importantly depends upon the strategies adopted by other individuals in the population [43, 44]. In cases where variation in life history strategies are affected by a relatively restricted set of supergenes, or major-effect genes, we expect NFDS to contribute to standing genetic variation.

Because mtDNA is haploid and maternally transmitted, its role as a supergene is in some ways distinct. Yet, the fact that it harbours a non-recombining block of key life history genes suggests that it can serve as a model for the fate of life history supergenes [24]. Interestingly, mtDNA often shows within-population polymorphism [30] and recent studies have revealed that much of this variation is in fact functional [31]. This implies that NFDS on mtDNA may be common, a possibility that is supported by both laboratory observations of allopatric haplotypes [41] and instances of geographically widespread sympatric coexistence between distinct mtDNA haplotype groups in other taxa (e.g., [45]). Needless to say, this has important ramifications for studies that implicitly or explicitly assume that mtDNA segregates as a neutral genetic marker [46].

In addition to the main effect of starting frequency, we also found support for an effect of environmental conditions on the strength of NFDS in our experiment. While this provides general support for a role of competition as a generator of NFDS, our expectation was to see stronger NFDS under more heterogeneous resource conditions in line with Lewontin’s [5] tenet. However, we found the opposite. In theory, the strength of NFDS should be collectively determined by the intensity of competition, the magnitude of G × E interactions for competitive ability and the degree of environmental heterogeneity. While our aim was to standardize net quantitative level of resources across food treatments, and to vary only the degree of heterogeneity, the observed population dynamics in our experimental evolution lines (Additional file 1) showed that this was not fully successful. Population size grew more rapidly and to a larger size in populations experiencing the more heterogeneous environment, implying that the absolute level of density dependent regulation was lower under these conditions at least during the first half of the experiement (Additional file 1). Previous experimental studies of other genes under NFDS in *Drosophila* have demonstrated that NFDS is weakened under less competitive conditions [26] and we suggest that the weaker NFDS seen in our heterogeneous environment may represent the result of less competitive conditions overall. We note, however, that alternative explanations include (i) the possibility that the equilibrium frequencies of haplotypes varied across food regimes, such that the equilibrium frequency of HI was considerably higher under the more heterogeneous environment, (ii) an impact of positive selection for HI in the the more heterogeneous environment and (iii) environment-specific complex mitonuclear epistatic effects (i.e., a G × G × E interaction [47]). In either case, this particular facet of our results does provide support for a functional link between the resource environment and the selective regime.

The main candidate gene responsible for the many documented functional differences between the sympatric HI and HII haplotype groups in *D. subobscura* (e,g., [32–36]) seems, remarkably, to not be a protein coding gene but rather a gene that affects translational events. The HI and HII haplotype groups show only two consistent differences (Additional file 1). One is a synonymous substitution within Nad5, which has served as the diagnostic basis for previous genotyping using restriction enzymes. The other is a SNP in 12S rRNA, located in a region homologous to the base of stem 15 in the mammalian 12S rRNA. Interestingly, the region in question is polymorphic in humans and there is evidence of both disease variants [48] and adaptive polymorphisms in 12S rRNA [49]. The specific HI/HII SNP in 12S rRNA of *D. subobscura* is associated with differences in the predicted secondary structure of the rRNA (Additional file 1), which may affect ribosomal protein synthesis and therefore also rate-dependent life history traits that are dependent upon the abundance of metabolic enzymes.

## Conclusions

Our work supports the view that we should consider balancing selection through NFDS a prime suspect among those processes that maintain genetic variation in life history genes in nature [3]. Moreover, the fact that ecological resources affected the NFDS regime in *D. subobscura* firmly places ecology at the root of maintenance of genetic variation of life history traits in this particular system. The emergence of NFDS on mtDNA may thus more generally be symptomatic of the fundamental and inevitable link between ecology and evolution [6–8, 43].

## Methods

### Construction of Mito-nuclear introgression lines

To isolate the genetic effects of mtDNA from the nuclear genome, our experiments were based on mitonuclear introgression lines (MNILs). The construction of our MNILs is explained in detail in Kurbalija Novicic et al. [32]. Briefly, all MNILs used were created from isofemale lines (IFLs), each of which was founded by a single wild-collected mated female originating from a common single natural population (Sicevo Gorge-Serbia, S 43°19′55.58″ N 22°0837.98″). Lines were first genotyped for their mtDNA haplotype using restriction enzymes [50] and we selected six IFLs, three of which carried HI haplotypes and three HII haplotypes, each of which was then backcrossed with a common seventh IFL (“D”). In each MNIL, backcrossing was performed by pairing 10 virgin females from a given IFL with 20 males from the D line. We used this repeated introgressive backcrossing procedure for 12 subsequent generations to replace the nuclear genome of a given IFL with the common outbred D nuclear genome (> 99.95% replaced). Note that IFLs were not inbred or otherwise made isogenic. To exclude the possibility of contamination during introgression, the mtDNA integrity of all MNILs were validated at generation 5, 8 and 12 by genotyping a sample of flies from each MNIL. We also screened for the presence of *Wolbachia* in all MNILs, by a PCR assay using 16S rDNA *Wolbachia*-specific primers [51] using methods detailed in García-Martínez et al. [37]*.* We used two different *Drosophila* strains containing *Wolbachia* as positive controls (*D. melanogaster* stock no. 5, Bloomington Stock Centre, *D. simulans*, Riverside strain). These PCR assays were negative for all of our MNILs as well as for the D line. All lines were maintained and all experiments performed under constant laboratory conditions, at 19 °C, 60% relative humidity, light of 300 lx, and at a photoperiod of 12 h light: 12 h dark.

### Founding of the experimental evolution populations

Prior to the experiments, we amalgamated the three MNILs carrying HI into one HI source population and the three HII MNILs into a HII source population to homogenize potential nuclear genetic variation across MNILs. This was done by mixing 100 adult flies from each MNIL, in two population cages (i.e., *N* = 3 × 100 flies per cage) (Plexiglas cages, L25 cm x W15 cm xH15 cm, with 3 dishes each containing 30 ml of cornmeal) and maintaining these for one subsequent full generation under standard laboratory conditions. The two source populations, thus, carried either the HI or the HII mtDNA haplotype, expressed in an outbred and common nuclear genetic background (i.e., D). Virgin flies from these two source populations were then sexed and used to found the experimental evolution populations.

We initiated *N* = 12 experimental evolution populations. In each population, *N* = 100 virgin flies (sex ratio 1:1) aged 3–5 days were introduced from the source populations into a population cage (L25 cm x W15 cm xH15 cm). We varied the starting frequency of HI and HII haplotypes and food resource conditions across populations, using a 2 × 2 crossed design (*N* = 3 populations per cell), in the following manner. In half of the cages, 80% of the founding flies were from the HI source population and 20% from the HII source population. In the other half, 20% of the founding flies were from the HI source population and 80% from the HII source population. In terms of food resource conditions, the amount of medium (both by volume and surface area) was identical in the two treatment groups while within population variation in nutrient concentration was manipulated as follows. Half of the cages (homogenous food conditions) contained 3 identical food dishes, each with 30 ml of standard cornmeal medium (YC) containing 1.5% of yeast. The other half of the cages (heterogeneous food conditions) also contained 3 dishes each with 30 ml standard medium, but these differed in yeast concentration (YL-0.375%, YC- 1.5%, YH- 6%).

### Maintenance of the experimental evolution populations

Populations were maintained in the laboratory in a manner that ensured discrete generations, 40 days/cycle [40], at 19 °C, 60% relative humidity and a 12 h light: 12 h dark cycle. At day 40, the three old food dishes were replaced by three new ones and flies were allowed in total 9 days for oviposition [40]. The new dishes, containing eggs and larvae, were then cleared from any adults and transferred to a new cage to start the next generation. All adult flies in each old cage were then counted, once they were dead.

### Sequencing and mitogenome assemblies

Prior the haplotype frequency estimation (see below), we sequenced and assembled the all mtDNA haplotypes used. DNA was extracted from the six IF lines (HI: 1, 3, 5; HII: 21, 25, 29) used to create the MNILs, using a salt-ethanol precipitation protocol. Flies were first gently macerated and placed in preparation buffer (100 mM NaCl, 10 mM Tris-HCl, pH = 8.0, 0.5% SDS) together with proteinase K, vortexed and incubated at 50 °C overnight. Samples were then frozen overnight. To precipitate DNA, we added saturated NaCl several times before adding 95% ethanol, and we spun the DNA into a pellet. The DNA pellet was suspended in TE 4 buffer (pH = 7.6). DNA quality and quantity was assessed using NanoDrop, Qubit and Bioanalyzer, followed by fragment length assessment on an agarose gel.

Sequencing libraries were prepared from 100 ng DNA using the TruSeq PCRfree DNA library preparation kit. The six samples were then sequenced to 125 bp paired-end reads in two lanes on an Illumina HiSeq2500 system using v4 sequencing chemistry. In total, we sequenced on average 194 million reads for each library. Mitogenomes from the six samples were then assembled using a 5% subset of the total number of reads from each library. Reads were fed to the MITObim V 1.8 algorithm [52] and the MIRA V 4.0.2 [53] assembler, to perform guided assemblies, using the *Drosophila pseudoobscura* mitogenome (GenBank: FJ899745.1) as a reference genome. All obtained assemblies were circular mitogenomes with a size of almost 16kbp. The final assemblies were then aligned using ClustalW [54] and MAFFT [55], and manually curated to obtain a final polished assembly for each haplotype. The assembled mitogenomes were annotated using DOGMA [56] and MITOS [57], using default parameter settings, and finally manually curated.

To assess the validity of our mitogenome assembly, all *Drosophila subobscura* mtDNA sequences available on GenBank (Cox1, Cox2, Cox3, Cob, Nad1, Nad2, Nad3, Nad5, rrnL, rrnS, the A + T rich region, and several trna), in total covering more than 50% of the total assembly, were aligned against our mitogenomes. Without exception, these showed > 99% sequence identity.

Several unique SNPs were found in each of the six mitogenome haplotype (see below), of which two consistently distinguished the HI and HII haplotype groups. Coverage depth for each SNP was verified by mapping back the reads used for the mitogenome assembly using Bowtie v 1.2 [58]. These efforts confirmed all SNPs identified in the assembly step. Here, we focus on the two main haplotype groups I and II, which show a striking and consistent pattern of within-population polymorphism across populations (Fig. 1) and functional phenotypic differences (see Introduction). Although SNPs do occur within each of the two haplotype groups, such SNPs are rare (e.g. [59]) and are not consistently polymorphic.

### Estimation of the mtDNA haplotype frequencies

We used pool-seq to estimate haplotype frequency evolution, by sequencing samples of flies from the 5th and 10th generation of each experimental evolution population. In each sample, 105 randomly selected flies per cage were pooled and subjected to DNA extraction (in groups of 15) and sequencing library preparations as described above. The *N* = 24 samples were then sequenced to 125 bp paired-end reads in two lanes on an Illumina HiSeq2500 system, using v4 sequencing chemistry. Our pool-seq effort was designed to provide sufficient sequencing depth for accurate estimation of mtDNA haplotype frequencies, but does not allow detailed analyses of the nuclear genome.

We sequenced, on average, 66 million reads for each library. Reads from each library were then mapped back to the six assembled mitogenomes, retaining only unique and zero-mismatch mappings using Bowtie v 1.2 [58]. The number of reads mapping to the two SNPs that distinguished the HI and HII types (in Nad5 and in 12S rRNA) were then counted and used as our estimate of the relative frequency of each main haplotype (HI or HII) in each sample.

### Statistical analyses

For each sample, all reads mapping to the two diagnostic SNPs (see below) were counted as being of either type HI or type HII. The proportion of HI reads for the two different SNPs were very closely correlated indeed across the 24 samples (*r* = 0.987), such that both markers provided virtually identical estimates. Here, we used the mean proportion across both SNPs here to estimate the frequency of HI present in each pool-seq sample.

Evolution can be defined as changes in genotype frequencies within a population, and our design allowed us to derive two temporally separated repeated measures of per generation haplotype frequency changes in our each evolving line as either Δ*f*_0–5_ = (*f*_5_ – *f*_0_)/5 or Δ*f*_5–10_ = (*f*_10_ – *f*_5_)/5 where subscripts denote the generation at which the sample was collected. In addition, we estimated Δ*f*_0–10_ = (*f*_10_ – *f*_0_)/10 to assess net haplotype frequency change. Because only two genotypes were involved, the frequency of HI: *f*_I_ = 1 – *f*_II_, and we thus restrict our analyses to changes in the frequency of one of the haplotypes. For each estimate of Δ*f*, we also derived a frequency dependent selection coefficient (*S*_I_) corresponding to the strength of selection required to cause the observed change in haplotype frequencies (see below).

Each evolving line represents an observational unit in our design, and we hence analysed our data using repeated measures ANOVAs (i.e., within-subjects ANOVAs). Here, each line represents the subject, and starting frequency of HI (0.2 or 0.8) and environmental condition (homogenous or heterogeneous) were two between-subjects factors, and the two repeated measures (of Δ*f* or *S*_I_) taken at different generational intervals was treated as a within-subjects factor. In these analyses, the focal between-subjects factors test for effects of our experimental treatments and the within-subjects factor tests whether the pattern of evolution changed during the course of our experiment. This analytical strategy draws on the fact that we track frequency dynamics in replicated and independent lines and the non-focal effect of random genetic drift, which is predicted to be substantial for mtDNA dynamics, forms a part of the residual term of our inferential models. Conventional *F*-tests of the between-subjects factors were validated using permutation tests, based on 9999 random permutations of data. Mean *S*_I_ for different groups were assessed using 95% CI’s from 9999 bootstrap replicates of data.

### Estimates of the strength of frequency dependent selection

We also wished to more explicitly assess whether the strength of frequency dependent selection on HI and HII differed across the two environmental experimental treatments (homogenous / heterogeneous). To allow this, we derived simple measures of frequency dependent selection from our empirical data using the following rationale. Previous explicit modelling of non-experimental data in this system has shown that the best fit with haplotype frequency dynamic data occurs where there is no positive or negative selection on these mtDNA haplotypes but where there is negative frequency dependent selection which is equally strong on both haplotypes [38]. We consider the two mtDNA haplotypes H_I_ and H_II_, with the frequencies *p*_*I*_ and *p*_*II*_ (*p*_*I*_ + *p*_*II*_ = 1) and fitnesses *W*_*I*_ and *W*_*II*_. The per-generation change in *p*_*I*_ is then given by

$$ \Delta  {p}_I=\frac{p_I{p}_{II}\left({W}_I-{W}_{II}\right)}{\overline{W}} $$

Observations from both natural populations (see Fig. 1; Additional file 1) and replicated laboratory cage populations [38, 40] also suggest that selection is symmetrical with an equilibrium for the two haplotypes H_I_ and H_II_ in the vicinity of *p*_*I*_ = *p*_*II*_ = 0.5. Assuming (i) an equilibrium frequency of *p*_*I*_ = *p*_*II*_ = 0.5, (ii) that haplotype fitness is linearly related to haplotype frequency and (iii) that selection is symmetrical, all of which are supported by previous studies [38, 40], we attain

$$ {W}_I=1-{p}_I{s}_I $$

$$ {W}_{II}=1-{p}_{II}{s}_I $$

where *s*_*I*_ is the frequency dependent selection coefficient. Given that $\overline{W}={p}_I{W}_I+{p}_{II}{W}_{II}$ we can then estimate *s*_*I*_ from our empirical observations of Δ*p*_*I*_ as

$$ {s}_I=\frac{-\Delta  {p}_I{p}_I-\Delta  {p}_I{p}_{II}}{-\Delta  {p}_I{p_{II}}^2-{p_I{p}_{II}}^2+{p_I}^2{p}_{II}-\Delta  {p}_I{p_I}^2} $$

Defined this way, *s*_*I*_ assumes an arbitrary scale but is positive when of Δ*p*_*I*_ changes towards the attractor and negative when it changes away from the attractor. For each cage, we estimated two independent repeated measures of *s*_*I*_ based on the observed haplotype frequency changes between generations 0–5 and 5–10, respectively. We note that our measure will be precise when the true equilibrium is near *p*_*I*_ = *p*_*II*_ = 0.5 but more approximate if the true equilibrium deviates from this condition.

## Supplementary information

**Additional file 1.** Supplementary results.

## Data Availability

All mitogenome assemblies have been deposited at Genbank (see Additional file 1). Raw sequencing data has been deposited at the NCBI Sequence Read Archive database (SRA) under the BioProject accession number PRJNA589517. Data from our experiments has been published in Mendeley Data and are available at DOI: 10.17632/59gxcyvzkc.1.

## Abbreviations

ATP: Adenosine triphosphate
G × E: Genotype by environment
IFL: Isofemale line
MNIL: Mitonuclear introgression line
mtDNA: Mitochondrial DNA
NFDS: Negative frequency dependent selection
OXPHOS: Oxidative phosphorylation
SNP: Single-nucleotide polymorphism
