# Supplementary material for: Negative frequency dependent selection contributes to the maintenance of a global polymorphism in mitochondrial DNA
Source: BMC Evol Biol. 2020 Feb 4;20:20. doi: 10.1186/s12862-020-1581-2 (PMC7001298; doi:10.1186/s12862-020-1581-2)
Supplement: Supplementary file 1 — Additional file 1. Supplementary results. [file 12862_2020_1581_MOESM1_ESM.pdf]

Negative frequency dependent selection maintains a global polymorphism in mitochondrial DNA

Zorana Kurbalija Novičić, Ahmed Sayadi, Mihailo Jelić and Göran Arnqvist

SI Results

Mitogenome organization. We sequenced and assembled six IFLs (three HI; three HII) of *Drosophila subobscura*. Mitogenome size range of IFLs was 15878-15879 bp. All assemblies are deposited in GenBank (NCBI/NIH) under the accession numbers MG421014, MG421015, MG421016, MG421017, MG421018, and MG421019.

All IFLs showed a gene order canonical for insects (SI Figure 1), with a high AT content (>78%). The IFL mitogenomes contain 22 tRNA genes, 13 protein-coding genes (PCGs), 16S and 12S ribosomal RNA, and an A+T region of 940 bp. The AT content of the A+T region is very high, more than 93%. PCG start and stop codons were identified based on the MITOS and DOGMA annotations and on the minimization of intergenic spaces and gene overlaps. All PCGs started with a standard initiation codon (ATN), except for Cox1 which was initiated by an (TCG) codon. Only Cox2 and Nad5 were terminated by an incomplete termination codon (T), while the rest of PCGs used (TAA) as a stop codon.

Previous experimental studies have shown that individuals carrying HI and HII differ in a wide range of life history traits, including metabolic rate, fertility, viability, longevity, desiccation resistance and fitness (e.g., Kurbalija Novicic et al. 2015, Castro et al. 2003; Christie et al. 2004; Jelić et al. 2015; García-Martínez et al. 1998; Fos et al. 1990). The two haplogroups HI and HII differ by a SNP located in Nad5 (Latorre et al. 1986, 1992; Alfonso et al. 1990; Castro et al. 1999, 2010). More specifically, among a larger set of restriction enzymes, one (HaeIII, which digests at GGCC or CCGG [5' and 3']) cuts HI in two locations, situated in Nad5 and rrnL, but HII in only rrnL. Our assemblies confirmed the SNP within Nad5 causing this divergent pattern of digestion. In addition, we identified a second SNP located in rrnS that distinguishes HI and HII haplotypes. Both of these SNPs were used to estimate HI/HII frequency evolution in our experimental evolution populations. Several other SNPs, unique to specific IFLs, were also found.

Across all 6 IFLs, there were in total 33 SNPs, 22 of which are synonymous and 6 of which are non-synonymous.

SI References

Afonso JM, Volz A, Hernández M, Ruttkay H, González M, Larruga JM, Cabrera VM and Sperlich D (1990) Mitochondrial DNA variation and genetic structure in Old-world populations of *Drosophila subobscura*. Mol Biol Evol. 7:123–42.

Castro JA, Barrio E, Gonzalez A, Picornell A, Ramon MM and Moya A (2010) Nucleotide diversity of a ND5 fragment confirms that population expansion is the most suitable explanation for the mtDNA haplotype polymorphism of *Drosophila subobscura*. Genetica 138:819–829.

Castro JA, Oliver P, Christie JS, Picornell A, Ramon M, Moya A (2003) Assortative mating and fertility in two *Drosophila subobscura* strains with different mitochondrial DNA haplotypes. Genetica 119:295–301.

Castro JA, Ramon M, Picornell A and Moya A (1999) The genetic structure of *Drosophila subobscura* populations from the islands of Majorca and Minorca (Balearic Islands, Spain) based on allozymes and mitochondrial DNA. Heredity 83:271.

Christie JS, Castro JA, Oliver P, Picornell A, Ramon MM, Moya A (2004) Fitness and life-history traits of the two major mitochondrial DNA haplotypes of *Drosophila subobscura*. Heredity. 93:371–378.

Fos M, Dominguez MA, Latorre A, Moya A (1990) Mitochondrial DNA evolution in experimental populations of *Drosophila subobscura*. Proc. Natl. Acad. Sci. U S A. 87:4198–4201.

García-Martínez J, Castro JA, Ramón M, Latorre A, Moya A (1998) Mitochondrial DNA haplotype frequencies in natural and experimental populations of *Drosophila subobscura*. Genetics 149:1377–82.

JelićM, Arnqvist G, Kurbalija Novičić Z, Kenig B, Tanasković M, Anđelković M, *et al.* (2015) Sex-specific effects of sympatric mitonuclear variation on fitness in *Drosophila subobscura*. BMC Evol. Biol. 15:135.

Kurbalija Novicic Z, Immonen E, Jelic M, Andjelkovic M, Stamenkovic-Radak M, Arnqvist G (2015) Within-population genetic effects of mtDNA on metabolic rate in *Drosophila subobscura*. J. Evol. Biol. 28:338–346.

Latorre A, Hernández C, Martínez D, Castro JA, Ramón M and Moya A (1992) Population structure and mitochondrial DNA gene flow in Old world populations of *Drosophila subobscura*. Heredity, 68:15–24.

Latorre A, Moya A and Ayala FJ (1986) Evolution of mitochondrial DNA in *Drosophila subobscura*. Proc. Natl. Acad. Sci. U S A. 83:8649–53.

SI Table 1. Repeated measures ANOVA of the effects of starting frequency (rare or common) and environmental conditions (homogenous or heterogeneous) on population size (log_{10} transformed) throughout the experiment. Generation represents each of the 10 generations during the experiment. See SI Figure 2.

Between-line effects:	df	MS	*F*	*P*
Starting frequency (SF)	1	0.10	2.15	0.181
Environment (E)	1	1.68	36.09	<0.001
SF × E	1	0.20	4.22	0.074
Residual	8	0.05		
Within-line effects:				
Generation (G)	9	0.27	20.07	<0.001
G × SF	9	0.01	0.57	0.819
G × E	9	0.04	3.17	0.003
G × SF × E	9	0.01	0.66	0.743
Residual	72	0.01		

Table S2. The absolute number of flies carrying mtDNA haplotype I, haplotype II or another haplotype group (i.e., other than I or II) in samples from natural populations of *Drosophila subobscura*. This compilation includes all samples found with N ≥ 10 isofemale lines or individual flies. See Figure 1.

Type I	Type II	Others	Locality	Source
12	10	1	Gävle, Sweden	Latorre et al 1992
12	16		Lilla leden, Sweden	Latorre et al 1992
10	10	2	Zurich, Switzerland	Latorre et al 1992
10	8	1	Cheste, Spain	Latorre et al 1992
4	7		Eureka, USA	Rozas et al. 1990
15	11		Gilroy, USA	Rozas et al. 1990
13	16		Santiago, Chile	Rozas et al. 1990
15	15	3	Barcelona, Spain	Rozas et al. 1990
1	2	61	Tenerife, Spain	Alfonso et al. 1990
2	10	2	Chechouan, Morocco	Alfonso et al. 1990
12	29	6	Ribeiro, Madeira	Alfonso et al. 1990
5	15		Poiso, Madeira	Alfonso et al. 1990
10	9		Villares, Spain	Alfonso et al. 1990
4	11	1	Escorial, Spain	Alfonso et al. 1990
4	9		Zurich, Switzerland	Alfonso et al. 1990
10	22	5	Tubingen, Germany	Alfonso et al. 1990
2	8		Scotland	Alfonso et al. 1990
1	11		Sweden (Uppsala, Gävle, Sundsvall)	Alfonso et al. 1990
4	5	16	La Gomera, Canary Islands	Pinto et al. 1997
6	8	17	La Palma, Canary Islands	Pinto et al. 1997
	40	2	El Hierro, Canary Islands	Pinto et al. 1997
	46	2	Grand Canaria, Canary Islands	Pinto et al. 1997
6	6	52	Tenerife, Canary Islands	Pinto et al. 1997
9	24	1	Morocco	Pinto et al. 1997
80	116	8	Esporles, Majorca	Garcia-Martinez et al. 1998
195	240	15	Valencia, Spain	Gonzales et al. 1994
95	106	24	Minorca, Spain	Castro et al. 1999
80	116	8	Majorca, Spain	Castro et al. 1999
272	311	24	Calvia, Spain	Christie et al. 2010
13	17		Old Seattle, WA, USA	Noor et al. 2000*
13	17	1	Seattle, WA, USA	Noor et al. 2000*
11	19		Mount St. Helena, CA, USA	Noor et al. 2000*
17	13		Provo, Utah, USA	Noor et al. 2000*
35	77	6	G, Serbia	Stamenković-Radak et al. 2012
15	34	6	BG, Serbia	Stamenković-Radak et al. 2012

35	28	6	DS, Serbia	Stamenković-Radak et al. 2012
16	44	2	DRG, Serbia	Stamenković-Radak et al. 2012
24	43	7	SG, Serbia	Stamenković-Radak et al. 2012
20	16	2	LRC, Serbia	Stamenković-Radak et al. 2012
				* Haplotype id inferred

Sources

Afonso JM, Volz A, Hernández M, Ruttkay H, González M, Larruga JM, Cabrera VM and Sperlich D (1990). Mitochondrial DNA variation and genetic structure in Old-world populations of *Drosophila subobscura*. Mol Biol Evol. 7:123–42.

Castro JA, Ramon M, Picornell A and Moya A (1999). The genetic structure of *Drosophila subobscura* populations from the islands of Majorca and Minorca (Balearic Islands, Spain) based on allozymes and mitochondrial DNA. Heredity 83:271-279.

Christie JS, Picornell A, Moya A, Ramon, MM & Castro JA (2010). Dynamics of the mtDNA haplotype variability in a *Drosophila subobscura* population over a two-year period. Open Evol. J. 4:23-30.

García-Martínez J, Castro JA, Ramón M, Latorre A and Moya A (1998). Mitochondrial DNA haplotype frequencies in natural and experimental populations of *Drosophila subobscura*. Genetics 149:1377-1382.

González A, Carrio R, Fernández-Pedrosa V and Moya A (1994). Lack of seasonal changes in mitochondrial DNA variability of a *Drosophila subobscura* population. J. Evol. Biol. 7:29-38.

Jelic M, Castro JA, Kurbalija Novicic Z, Kenig B, Dimitrijevic D, Savic Veselinovic M and Andjelković M (2012) Absence of linkage disequilibria between chromosomal arrangements and mtDNA haplotypes in natural populations of *Drosophila subobscura* from the Balkan Peninsula. Genome 55:214–221.

Latorre A, Hernández C, Martinez D, Castro JA, Ramón M and Moya A (1992). Population structure and mitochondrial DNA gene flow in Old World populations of *Drosophila subobscura*. Heredity 68:15-24.

Noor MA, Pascual M and Smith KR (2000) Genetic variation in the spread of *Drosophila subobscura* from a nonequilibrium population. Evolution, 54:696-703.

Pinto FM, Brehm A, Hernandez M, Larruga JM, González AM and Cabrera VM (1997) Population genetic structure and colonization sequence of *Drosophila subobscura* in the Canaries and Madeira Atlantic islands as inferred by autosomal, sex-linked and mtDNA traits. J. Hered. 88:108-114.

Rozas J, Hernandez M, Cabrera V and Prevosti A (1990). Colonization of America by *Drosophila subobscura*: Effect of the Founder Event on the Mitochondrial DNA Polymorphism. Mol. Biol. Evol. 7:103-109.

Stamenković-Radak, M, Jelic M, Novičič, ZK, Kenig B, Tanaskovic M and Andjelković, M (2012) Balkan glacial history and modern *Drosophila subobscura* population genetics. Evol. Ecol. Res. 14:839-858.

Table S3. Observed mtDNA haploype frequencies in the 12 laboratory populations at generation 5 and 10 of the experimental evolution.

**Cage ID**	**Environment**	**Generation**	**Starting frequencies (HI:HII)**	**HI**	**HII**
OV1-7	homo	5	80: 20	0.4096	0.5904
OV8-14	homo	5	80: 20	0.83365	0.16635
OV15-21	homo	5	80: 20	0.83595	0.16405
OV22-28	homo	5	20: 80	0.2041	0.7959
OV29-35	homo	5	20: 80	0.56935	0.43065
OV36-42	homo	5	20: 80	0.2688	0.7312
OX1-7	homo	10	80: 20	0.6806	0.3194
OX8-14	homo	10	80: 20	0.16255	0.83745
OX15-21	homo	10	80: 20	0.72845	0.27155
OX22-28	homo	10	20: 80	0.6164	0.3836
OX29-35	homo	10	20: 80	0.77045	0.22955
OX36-42	homo	10	20: 80	0.1636	0.8364
HV1-7	hetero	5	80: 20	0.7066	0.2934
HV8-14	hetero	5	80: 20	0.66055	0.33945
HV15-21	hetero	5	80: 20	0.9981	0.0019
HV22-28	hetero	5	20: 80	0.21985	0.78015
HV29-35	hetero	5	20: 80	0.4161	0.5839
HV36-42	hetero	5	20: 80	0.44135	0.55865
HX1-7	hetero	10	80: 20	0.66055	0.33945
HX8-14	hetero	10	80: 20	0.9033	0.0967
HX15-21	hetero	10	80: 20	0.9986	0.0014
HX22-28	hetero	10	20: 80	0.3103	0.6897
HX29-35	hetero	10	20: 80	0.5219	0.4781
HX36-42	hetero	10	20: 80	0.6634	0.3366

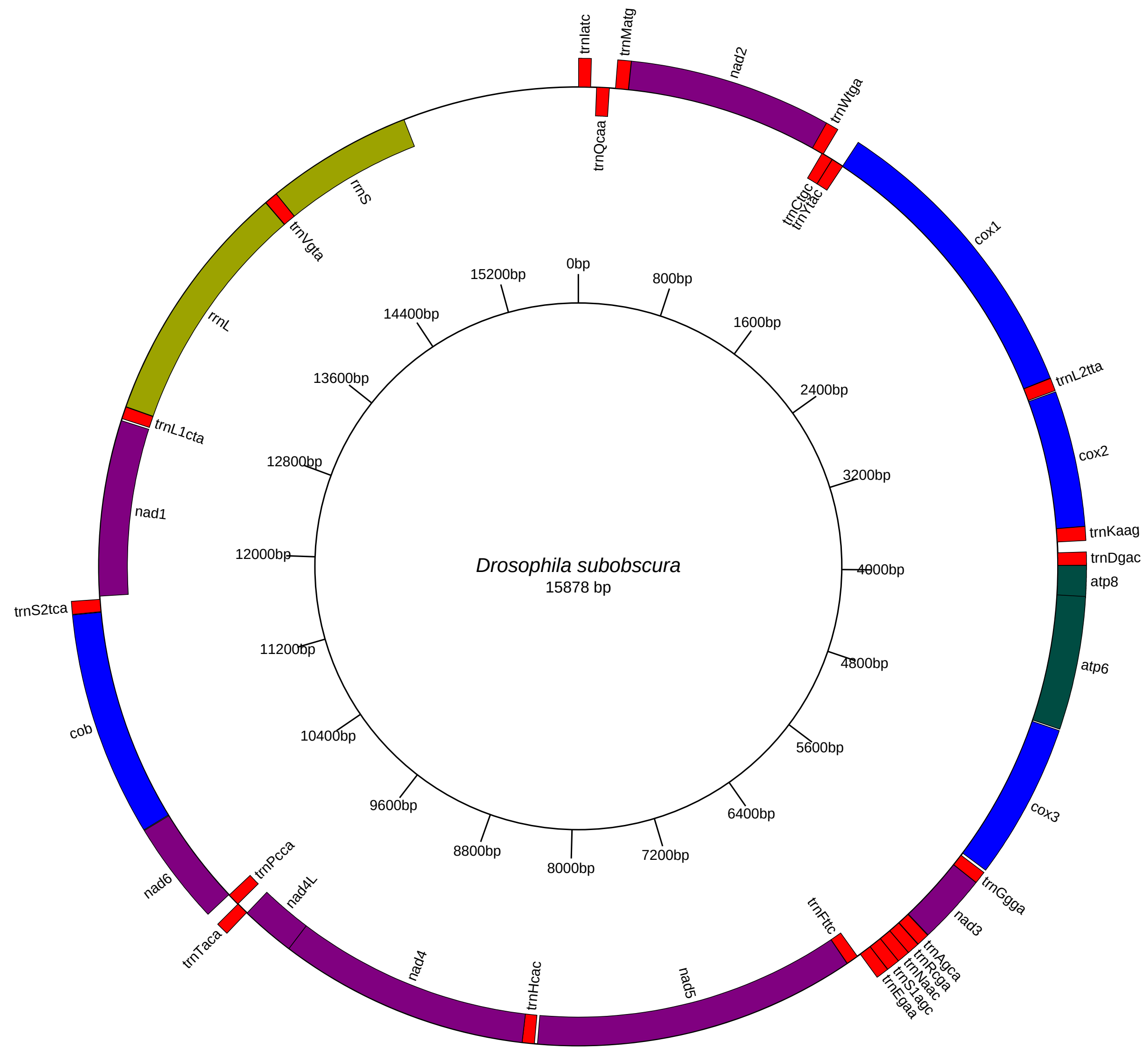

SI Figure 1. *Drosophila subobscura* mitogenome organisation.

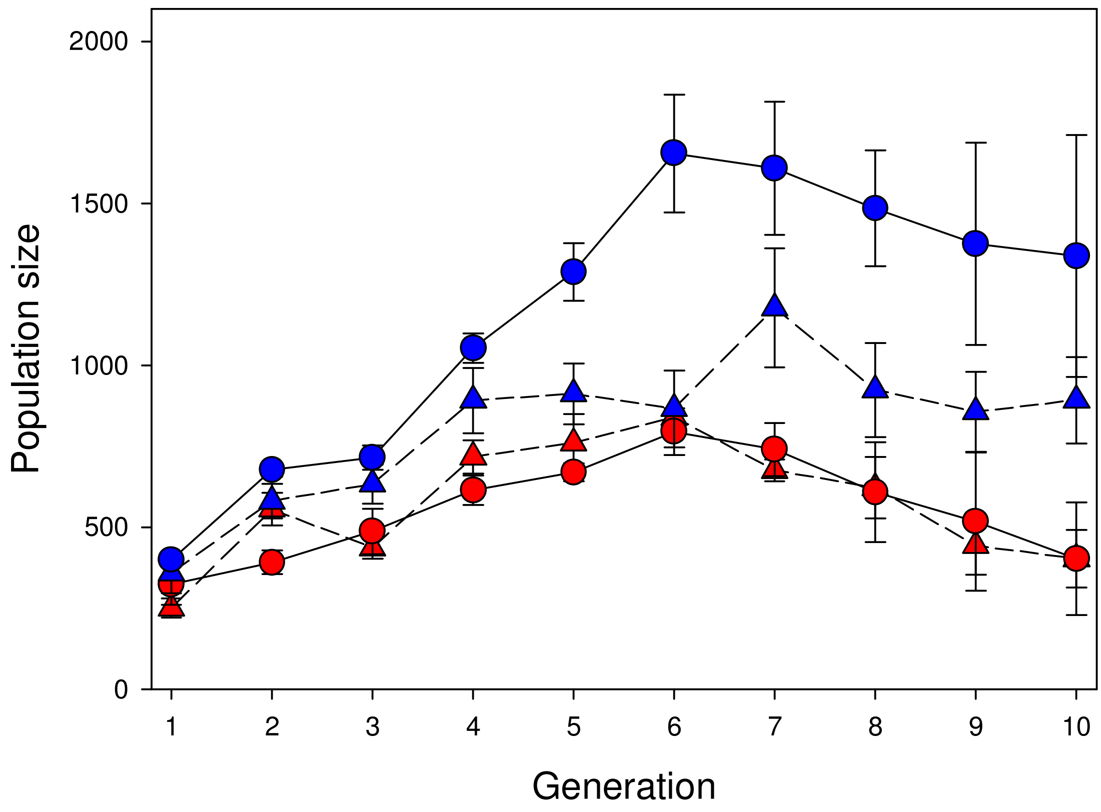

SI Figure 2. Mean observed population size (±SE) in cage populations throughout the experiment. Blue symbols denote the heterogeneous environment, red homogenous, triangles and dashed lines populations where HI was started as common and circles and solid lines populations where HI was started as rare. Cage populations experiencing a heterogeneous environment reached an overall higher population size, but the starting frequency of haplotypes had no significant effects on population size (SI Table 1).

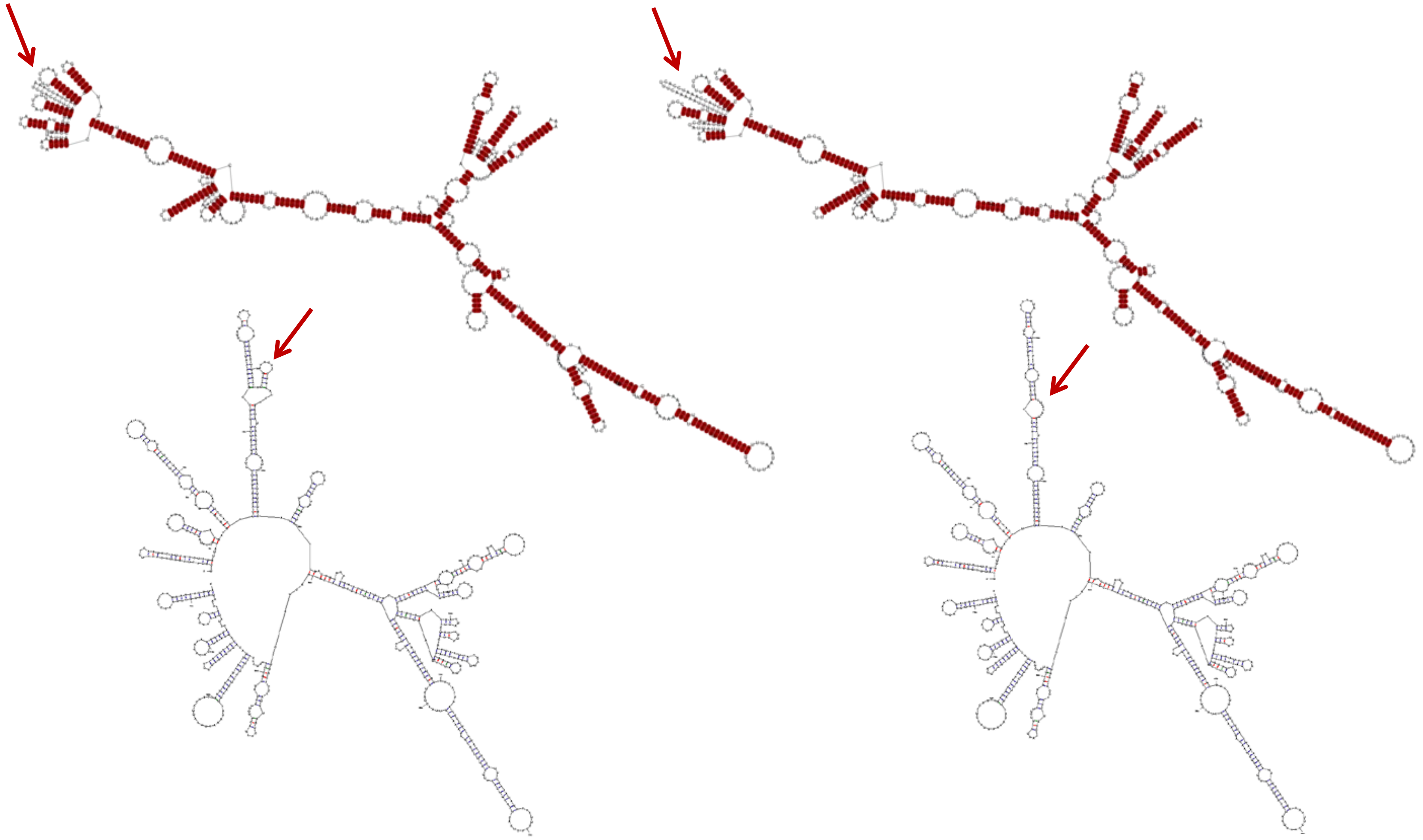

SI Figure 3. Predicted secondary RNA structure of 12S rRNA of HI (left) and HII (right), based on folding in LocARNA (top) and mfold (bottom). Folding was performed using default settings, apart from folding temperature which was set to 21°C. Predicted structural differences between the two haplotypes are highlighted with red arrows.
